# Gamma‐secretase inhibitor suppressed Notch1 intracellular domain combination with p65 and resulted in the inhibition of the NF‐κB signaling pathway induced by IL‐1β and TNF‐α in nucleus pulposus cells

**DOI:** 10.1002/jcb.27504

**Published:** 2018-10-26

**Authors:** Yao Huang, Wei Mei, Jian Chen, Tao Jiang, Zheng Zhou, Guoyong Yin, Jin Fan

**Affiliations:** ^1^ Department of Orthopaedics Institute of Sport Medicine, Affiliated Hospital of Nanjing University of TCM Nanjing China; ^2^ Department of Orthopaedics Institute of Traumatology Department, Affiliated Hospital of Nanjing University of TCM Nanjing China; ^3^ Department of Orthopaedics The First Affiliated Hospital of Nanjing Medical University Nanjing China; ^4^ Department of Orthopedics Wuxi People's Hospital Affiliated to Nanjing Medical University Wuxi China

**Keywords:** NF‐κB, notch, notch1 intracellular domain, nucleus pulposus cell, p65

## Abstract

In this experiment, the cross‐talk betweenNotch and the NF‐κB signaling pathway was examined to reveal the mechanism of slowing down the type II collagen (ColII) and aggrecan degeneration affected by inflammatory cytokines. The expression levels of ColII and aggrecan in the intervertebral disc were observed through immunohistochemistry and hematoxylin‐eosin staining+alcian blue staining, respectively. The expression levels of ColII, aggrecan, Runx2, and NF‐κB in the nuclei of human nucleus pulposus cells (hNPCs) in each group, as well as the phosphorylation and acetylation levels of p65, were examined through Western blot analysis. The 293T cells were transfected with a plasmid containing the overexpressed relative domain of Notch1 intracellular domain (NICD1), and immunoprecipitation (IP) was performed to observe the combination of NICD1 and p65. HNPCs were transfected with a lentiviral‐contained overexpression lacking the ANK region of NICD1, and IP was performed to observe the combination of NICD1 and p65. The expression of ColII and aggrecan in the intervertebral disc culture increased when γ‐secretase inhibitor N‐[N‐(3,5‐difluorophenacetyl)‐1‐alanyl]‐Sphenylglycine t‐butyl ester (DAPT) was added to the disc culture medium. Western blot revealed that DAPT inhibited p65 phosphorylation and acetylation, and the p65 and p50 levels in the nucleus decreased. NICD1 was found to be combined with p65 in contrast to the reverse consequences after ANK domain deletion in hNPCs. In nucleus pulposus cells, the combination of p65 and the ANK domain of NICD1 is a critical procedure for the degeneration related to the NF‐κB signaling pathway activation induced by IL‐1β and TNF‐α.

## 1 INTRODUCTION

Approximately 84% of the world's population experiences lower back pain at a certain time, and approximately 10% suffer from chronic disabilities.^1^ As a disease with the second‐highest incidence, lower back pain has considerably affected the quality of life and caused a large economic burden. The most common cause of lower back pain is intervertebral disc degeneration.^2^ An in‐depth understanding of the etiology of intervertebral disc degeneration is beneficial for the optimal treatment of intervertebral disc degeneration and the development of new drugs to combat disc degeneration.

Intervertebral disc degeneration is characterized by the expression of type II collagen (ColII) and protein polysaccharides in the intervertebral disc, thereby reducing the elasticity modulus of the intervertebral disc as well as causing the loss of water and a decrease in altitude. Inflammatory factors play an important role in intervertebral disc degeneration, which leads to pathological reactions, such as matrix degradation, cell senescence, apoptosis, and nerve and blood vessels entering, thereby inducing severe degeneration and pain in the intervertebral disc.^3^

The significant increase in the expressions of inflammatory factors and matrix metalloproteinases in the intervertebral disc is closely related to the activation of the NF‐κB signaling pathway, and these substances are the downstream target genes of NF‐κB signaling pathways.^4^ The activation of this pathway in nucleus pulposus cells of degenerative intervertebral disks is significantly higher than that in a normal intervertebral disc nucleus.^5^ Therefore, in studies on intervertebral disc degeneration, the abnormal activation of this pathway should be regulated; this regulation consequently inhibits such an abnormal activation in a mouse intervertebral disc degeneration model and significantly increases the expression of ColII and aggrecan protein in the intervertebral disc.^6^ In an organ culture of ox bone intervertebral disc, the release of matrix metalloproteinases significantly decreases after the NF‐κB signaling pathway is inhibited.^7^

Notch is a single transmembrane receptor protein that can widely regulate cell life. This protein is composed of three parts, namely, the extracellular domain, transmembrane region, and cell area, and each part plays different roles that determine the function of a Notch receptor protein. Mammals have four species of Notch receptors, including Notch1, Notch2, Notch3, and Notch4. When a Notch receptor is activated, it is cleaved by γ‐secretase enzymes, released into the intracellular region, and transferred to the nucleus. A known Notch‐specific inhibitor is N‐[N‐(3,5‐difluorophenacetyl)‐1‐alanyl]‐Sphenylglycine t‐butyl ester DAPT.^8^

The correlation between the Notch signaling pathway and inflammation has been observed, and studies have shown that many inflammatory diseases, such as rheumatoid arthritis,^9^ systemic lupus erythematosus,^10^ atherosclerosis,^11^ primary cholestasis, and bacterial or viral infection,^12^ occur when the Notch signaling pathway is active. Therefore, we would like to determine whether the secretion of proinflammatory factors and the activation of the inflammatory pathway are related to the Notch signaling pathway in intervertebral disc degeneration and whether the inflammation can be controlled through the Notch signaling pathways.

In this study, mouse intervertebral disks and human nucleus pulposus cells (hNPCs) were investigated to establish degeneration models. Our results confirmed that numerous activation sites were found in the NF‐κB signaling pathway, the activation of the NF‐κB signaling pathway was difficult to effectively inhibit by single site inhibitors, and activation sites in the Notch signaling pathway were relatively single. Considering that DAPT can suppress the entire pathway, we hypothesized that NF‐κB and Notch signaling pathways were implicated in the degeneration of nucleus pulposus cells, and the Notch signaling pathway was activated after DAPT inhibited the NF‐κB pathway. The mechanism of DAPT in the degeneration of nucleus pulposus cells under the action of inflammatory factors was also explored.

## 2 MATERIALS AND METHODS

### 2.1 Animals

Male B6 mouse (6 weeks of age) was obtained from the Model Animal Research Center of Nanjing University (Nanjing, China). Animals were maintained at room temperature with free access to water and food. All the experimental procedures were performed with the approval of the Experimental Research Institute of the First Affiliated Hospital of Nanjing Medical University.

### 2.2 Cell and mouse intervertebral disks treatment

Four groups of a mouse intervertebral disks organ culture and hNPCs were prepared: Normal group, Degeneration group: IL‐1β (10 ng/mL; PeproTech, Rocky Hill, New Jersey)+TNF‐α (50 ng/mL; PeproTech, Rocky Hill, NJ), ITDL group: IL‐1β (10 ng/mL)+TNF‐α (50 ng/mL)+DAPT (5 μmol/mL, MCE; New Jersey), ITDH group: IL‐1β (10 ng/mL)+TNF‐α (50 ng/mL)+DAPT (20 μmol/mL). The mouse intervertebral disc was cultivated for 3 and 10 days.

### 2.3 Plasmids and lentiviral particle transduction

Plasmids with overexpression of NICD structural domain were purchased from GENECHEM Company (Shanghai, China; Table 1). The 293T cells were seeded in 10‐cm plates (1.3 × 10^6^ cells/plate) in 1640 medium (Gbico, Grand Island, New York) with 10% heat‐inactivated fetal bovine serum (FBS) 2 days before transfection. After 16 hours, the transfection medium was removed and replaced with 1640 medium with 5% heat‐inactivated FBS and penicillin/streptomycin. Cells were harvested for protein extraction 5 days after plasmids transduction. Lentiviral particles with overexpression of RAM+translation active domain (TAD)+PEST structural domain were purchased from GENECHEM Company. HNPCs were seeded in 10‐cm plates (1.0 × 10^6^ cells/plate) in medium one day before transfection. After 24 hours, conditioned medium was removed and replaced with 4801 medium. Cells were harvested for protein extraction 6 days after viral transduction.

**Table 1:** Primer sequences

Gene		Primer sequence
Col2a1	Sense	5′TGGACGATCAGGCGAAACC 3′
	Anti‐sense	3′GCTGCGGATGCTCTCAATCT 5′
AGC1	Sense	5′ACTCTGGGTTTTCGTGACTCT 3′
	Anti‐sense	3′ACACTCAGCGAGTTGTCATGG 5′
Col 10a1	Sense	5′GGGGCTAAGGGTGAAAGGG 3′
	Anti‐sense	3′GGTCCTCCAACTCCAGGATCA 5′
Runx2	Sense	5′TGGTTACTGTCATGGCGGGTA 3′
	Anti‐sense	3′TCTCAGATCGTTGAACCTTGCTA 5′
GAPDH	Sense	5′GCACAGTCAAGGCCGAGAAT 3′
	Anti‐sense	3′GCCTTCTCCATGGTGGTGAA 5′

### 2.4 Culture of mouse intervertebral disks

For the organ culture of mouse intervertebral disks, mice under carbon dioxide anesthesia were killed by cervical dislocation and soaked in 75% alcohol for 5 minutes before sterile operation on the super clean bench. Segments were removed from the lumbar spines of the mice with back‐cuts. The intervertebral disks L2‐3 and L3‐4 (including the upper and lower endplates) were dissected and cultured on a 6‐pore plate, with 4 mL of the culture medium added to each plate. The mouse intervertebral disks were cultured and placed in an incubator containing 5% CO_2_ and at 37°C, with the medium changed every other day. The culture media for the mouse intervertebral disks contained 10% FBS (Hyclone, Logan City, Utah), 1% double‐antibody (streptomycin+penicillin; Gibco, Grand Island, NY), and 1:1 DMEM/F12 (Hyclone, South Logan, UT) in the volume fraction.

### 2.5 Cell culture

hNPCs (ScienCell 4800, Carlsbad, CA) and cell‐specific media (4801) were purchased from ScienCell. hNPCs were cultured at 37°C/5% CO_2_, and then grown to 90% confluence. HNPCs within the five passages of subculture were then collected for the subsequent experiments. The 293T cells were purchased from Chinese Academy of Sciences cell bank (Shanghai, China). The cells were cultured with 1640 medium (Gbico) contained 10% fetal bovine serum (Hyclone, Utah), 1% double‐antibody (streptomycin+penicillin; Gibco).

### 2.6 Immunohistochemistry

For the ColII immunohistochemistry, The paraffin sections were de‐waxed, dehydrated, and incubated overnight at 4°C with anti‐ColII (diluted by 1:100; Abcam, Cambridge, UK). After the primary antibody was removed, the secondary antibody (diluted by 1:100; Thermo, Waltham, MA) was added; and, the sections after being incubated for 1 hour at room temperature. The stained cells were developed with diaminobenzidine, counterstained with hematoxylin.

### 2.7 Nuclear protein extraction and Western blot analysis

Cells were placed on ice immediately following treatment and washed with ice‐cold Hank's balanced salt solution. Nuclear proteins were prepared using the CellLytic NuCLEAR extraction kit (Sigma‐Aldrich, St. Louis, MO). All the wash buffers and final resuspension buffer included 1× protease inhibitor cocktail (Pierce, Rockford, IL), NaF (5 mM), and Na3VO4 (200 mM). The protein concentration of the lysate was measured using the BCA protein assay kit (Thermo Fisher Scientific, Boston, MA). Nuclear or total cell proteins were resolved on 8% to 12% sodium dodecyl sulfate polyacrylamide gel electrophoresis and transferred by electroblotting to nitrocellulose membranes (Bio‐Rad, Hercules, CA). The membranes were blocked in nonfat dry milk solution and incubated overnight at 4°C with ColII (1:1000), aggrecan (1:1000), Runx2 (1:1000), ColX (1:1000), p65 (1:1000), p50 (1:1000), p52 (1:500), RelB (1:500), cRel (1:1000), and NICD1 (1:1000) primary antibody dilution buffer and then incubated with horseradish peroxidase‐conjugated anti‐rabbit IgG (1:5000) for 2 hours. Afterwards, the membranes were developed using the enhanced chemiluminescence substrate LumiGLO (Millipore, Bedford, MA) and exposed to X‐ray film. The bands were analyzed with Gel‐Pro Analyzer 4.0.

### 2.8 Immunoprecipitation

For immunoprecipitation (IP), the cells were cultured in a 10 cm cell culture dish, and a precooled IP lysis liquid was added to digest the cells in an EP tube at 4°C. The resulting mixture was then centrifuged at 14 000*g* for 15 minutes. The supernatant was collected via the Bradford method, and a protein standard curve was plotted. The protein concentration was also determined. The same mass protein was obtained and mixed at the same volume after the supernatant was transferred. An antibody was added according to a volume‐to‐volume ratio of 100:1 and at 4℃. The container with the obtained mixture was inverted and incubated for 2 hours. Afterward, 5 μL of protein A/G magnetic beads were added at 4°C. The container with these beads was also placed upside down and incubated overnight. The supernatant was removed from the magnetic rack, and the magnetic bead was cleaned. Subsequently, 40‐60 μL of the loading sample buffer solution was added and boiled for 10 minutes. The liquid was removed from the magnetic rack, loaded, and stored at −80°C for electrophoresis.

### 2.9 RNA isolation and quantitative reverse transcription‐polymerase chain reaction

Primers for real time polymerase chain reaction (PCR) listed in Table 1 were designed using Primer‐BLAST (http://www.ncbi.nlm.nih.gov/tools/primer‐blast/). Total RNA from hNPCs was isolated with Trizol® Reagent (Invitrogen, Carlsbad, CA), and cDNA was synthesized by First Strand cDNA Synthesis Kit (Thermo, Waltham, MA) according to the manufacturer's instructions. Quantitative reverse transcription‐PCR (qRT‐PCR) was performed by using iQ5 optical system software (Bio‐Rad Laboratories, Hercules, CA) with Fast Start Universal SYBR Green Master (ROX; Recho, Basel, Switzerland) for mRNA quantitation of all referred genes. Relative expression was calculated using the 2^−ΔΔCt^ method normalized to GAPDH (endogenous loading control).

### 2.10 Statistical analysis

SPSS 19.0 statistical software (SPSS, Inc, Chicago, IL) was used for all statistical analysis. The measurements were presented as mean ± standard deviation, and data were analyzed using one‐way analysis of variance followed by a Bonferroni's post‐hoc test for multiple comparisons. *P* < 0.05 was considered to indicate a statistically significant difference.

## 3 RESULTS

### 3.1 DAPT attenuated the degeneration of nucleus pulposus cells induced by IL‐1β and TNF‐α in a mouse intervertebral disc organ culture

**Figure 1:**
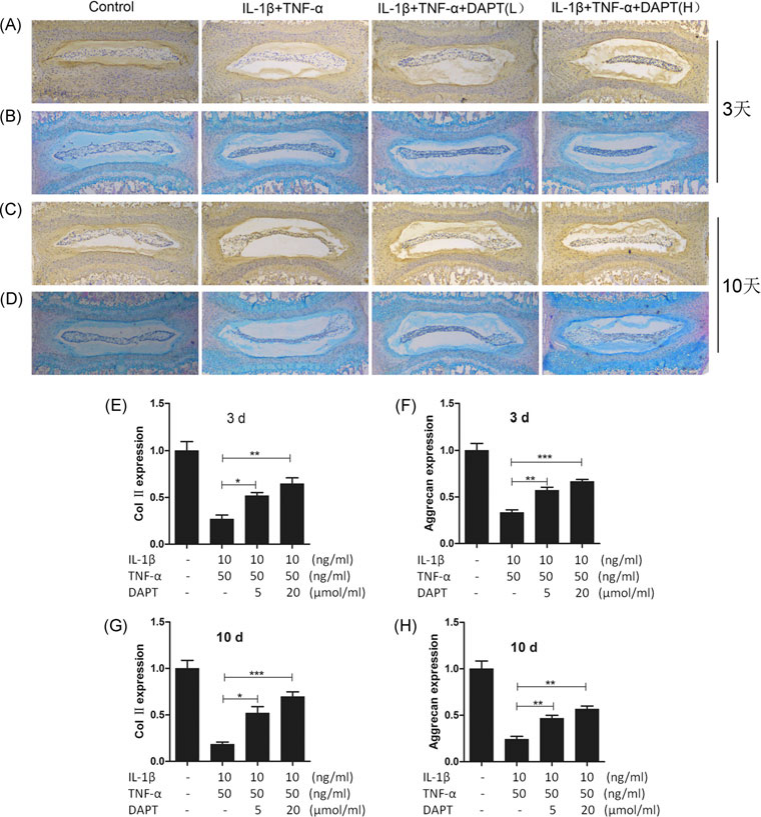
The protein expression of Col II and aggrecan in mouse discs. A, Col II immunohistochemistry of mouse intervertebral disc culture in 3 days; B, 3‐day aggrecan HE + Alcian blue staining in mouse intervertebral disc culture; C, Col II immunohistochemistry of mouse intervertebral disc culture in 10 days; D, 10‐day aggrecan HE + Alcian blue staining in mouse intervertebral disc culture; Original magnification, 100×. E, statistical chart of Col II expression in immunohistochemistry; F, Statistical chart of the expression of aggrecan from a protein stained with HE + Alcian blue; G, Statistical chart of Col II expression in immunohistochemistry; H, Statistical chart of the expression of aggrecan from a protein stained with HE + Alcian blue. *P > 0.05, **P > 0.01, ***P > 0.001

The effect of DAPT on a degeneration model using a mouse intervertebral disc organ culture was evaluated according to previously described methods.^13^ The content of ColII in the intervertebral disc of the induced degeneration group decreased significantly, and the content of ColII increased as the concentration of DAPT in the culture medium increased. Immunohistochemical staining revealed a significantly increased ColII content after DAPT was added to the degenerative group in Figure 1A and 1C. ColII and aggrecan in nucleus pulposus cells must be secreted to maintain the physiological function of the intervertebral disc. As such, we determined the effect of DAPT on the secretion of aggrecan in the degenerated intervertebral disks. The secretion of aggrecan was inhibited in the degenerative group. Such inhibition was reduced, and the distribution of aggrecan was increased in the DAPT group (Figure 1B and 1D). A statistical histogram showed a statistically significant increase in the ColII and aggrecan distribution (Figure 1E‐H).

### 3.2 Effects of DAPT on the IL‐1β and TNF‐α expression induced in hNPCs in relation to intervertebral disc degeneration‐associated proteins and transcription factors

**Figure 2:**
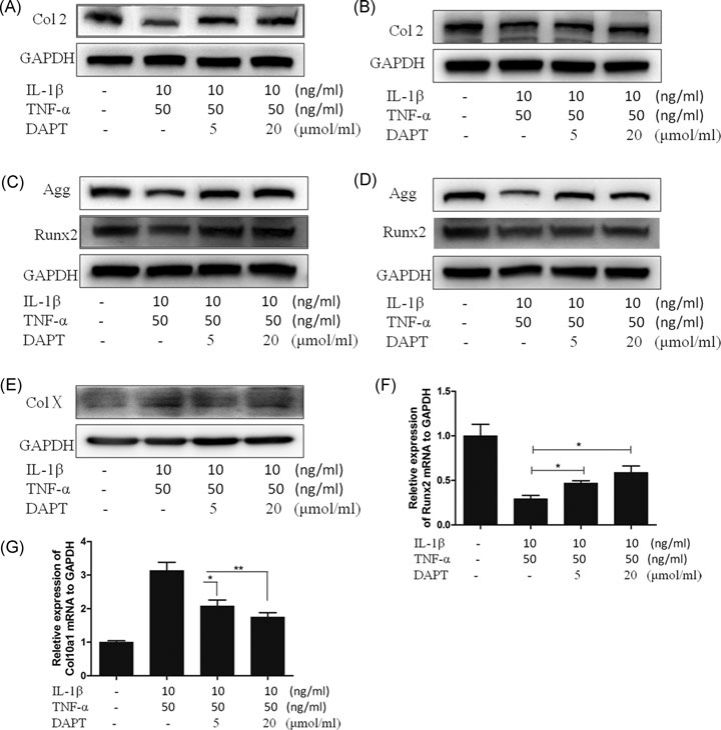
The protein expression of ColII, aggrecan, Runx2 and ColX in hNPCs. A, Western blot of ColII in 24 hours of hNPCs. B, Western blot of ColII in 48 hours of hNPCs. C, Western blot of aggrecan and Runx2 in 24 hours of hNPCs. D, Western blot of aggrecan and Runx2 in 48 hours of hNPCs. E, Western blot of ColX in 48 hours of hNPCs. F and G, qRT‐PCR for mRNA expression of Runx2 and ColX in 48 hours (normalized to GAPDH). *P < 0.05, **P < 0.01. ColII, type II collagen; hNPCs, human nucleus pulposus cells

Nucleus pulposus cells are chondrocytes, and their secretion of ColII and aggrecan is consistent with the mechanism of chondrocyte regulation. Their secretion in mature nucleus pulposus cells is regulated by Runx2. In our study, we detected ColII and aggrecan levels secreted by hNPCs at 24 and 48 hours and found that ColII and aggrecan were significantly inhibited by IL‐1β and TNF‐α at these time points. The inhibition of the expression rate was reduced after DAPT was added. ColII and aggrecan secretion levels increased in a concentration‐dependent manner (Figure 2A‐D). We also showed that the expression pattern of the regulatory factor of Runx2 was consistent with those of ColII and aggrecan (Figure 2C and 2D). IL‐1β and TNF‐α induce hNPC degeneration, indicating the aging of hNPCs. As such, we determined the expression level of hNPCs at 48 hours and observed that IL‐1β and TNF‐α induced the expression of ColX, but this expression decreased after DAPT was added, suggesting that DAPT could antagonize hNPC degeneration induced by IL‐1β and TNF‐α (Figure 2E). We further examined the transcription factors of Runx2 and ColX and revealed that they were consistent with their protein expression levels (Figure 2F and 2G).

### 3.3 Suppression of IL‐1β‐ and TNF‐α‐induced phosphorylation and acetylation of p65 by DAPT

**Figure 3:**
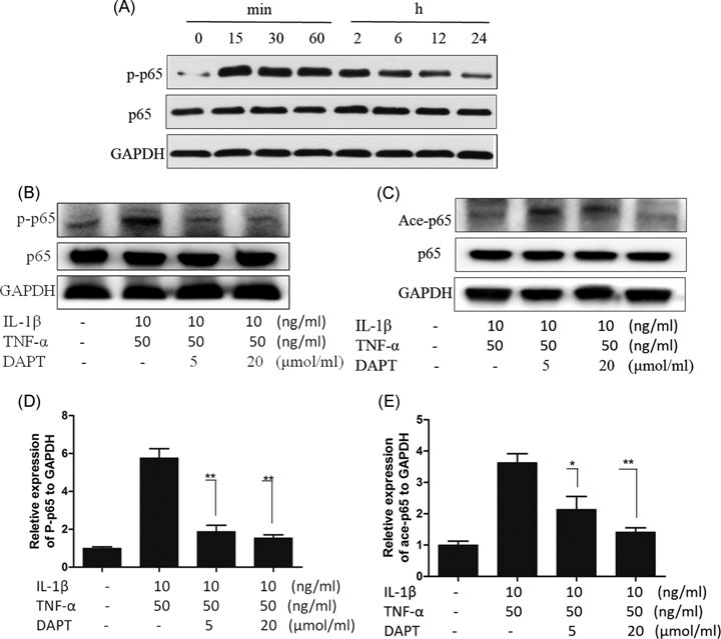
Phosphorylation and acetylation of p65 in hNPCs. A, Western blot of p65 phosphorylation level of the culture medium at different time points after adding TNF‐α (50 ng/ml); B and C, Western blot of p65 phosphorylation and acetylation of hNPCs. D and E, Statistical chart of the p65 phosphorylation and acetylation. *P > 0.05, **P > 0.01

The main mechanism of the classical activation of the NF‐κB signaling pathway involves the release and nucleation of the p65‐p50 dimer. After p65 is phosphorylated, p65‐p50 translocates into the nucleus, and p65 acetylation determines the activation level of the p65‐p50 dimer in the nucleus. The acetylated p65 moves out of the nucleus and forms a stable trimer with IκB and p50, resulting in the deactivation of the p65‐p50 dimer signaling pathway. In our TNF‐α stimulation, the p65 phosphorylation level in hNPCs was high within 15 minutes to 6 hours (Figure 3A). In previous experiments, DAPT inhibition and NICD1 shearing and nucleation at 2 hours is evident in hNPCs. Therefore, we examined the IL‐1β and TNF‐α‐induced Notch and NF‐κB signaling pathways at 2 hours and detected the phosphorylation and acetylation levels of p65 at the same time point. Our results demonstrated that with IL‐1β and TNF‐α induction, the p65 phosphorylation and acetylation levels significantly declined after DAPT was added (Figure 3B‐E). The high‐concentration DAPT suppression was obvious, indicating that DAPT inhibits the activation of the NF‐κB signaling pathway by blocking the classical NF‐κB p65 pathways.

### 3.4 Suppression of IL‐1β‐ and TNF‐α‐induced nucleation of p65, p50, p52, and RelB BY DAPT

**Figure 4:**
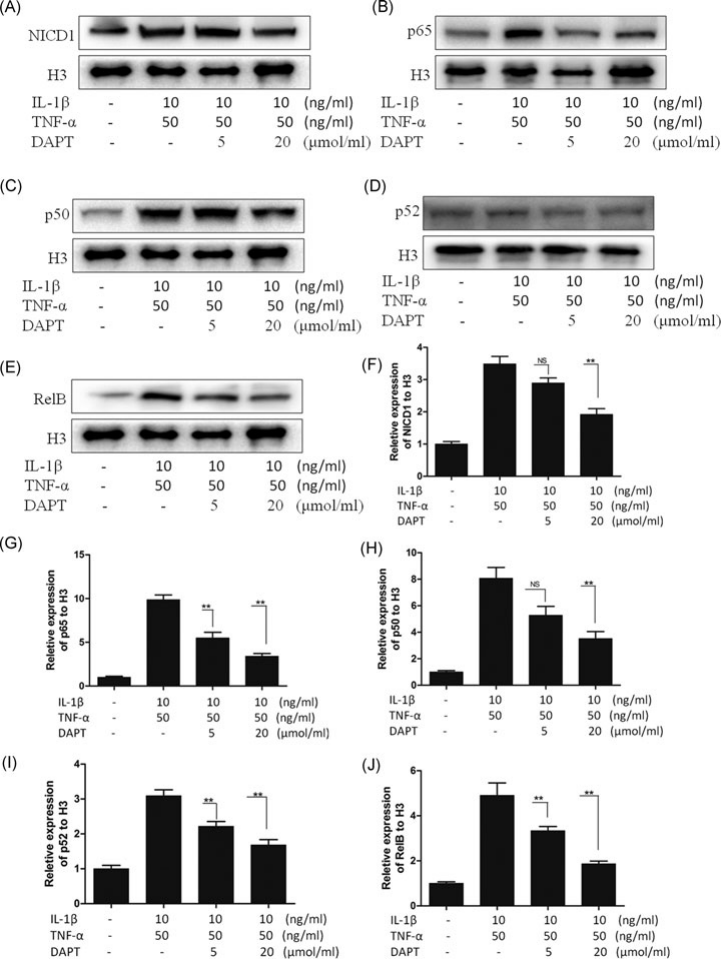
A‐E, Western blot for the nuclear level of NICD1, p65, p50, p52, and RelB in 2 hours of adding IL‐1β (10 ng/mL)+TNF‐α (50 ng/mL). F‐J, Statistical chart of the nuclear expression of NICD1, p65, p50, p52, and RelB. *P < 0.05, **P < 0.01. NS, no significance

In an activated NF‐κB signaling pathway, the corresponding target gene is stimulated by five subunits, and each subunit forms a homologous or heterologous dimer in the nucleus. P65 phosphorylation is related to the dimer nucleation associated with p65, whereas p65 acetylation is linked with the dimer out of the nucleus associated with p65. The activation level of the NF‐κB signaling pathway is also correlated with the level of the NF‐κB subunits in the nucleus. As such, we examined the levels of the NF‐κB subunits in the nucleus and found that the expression levels of p65, P50, P52, and RelB were significantly higher during IL‐1β and TNF‐α induction at 2 hours. In comparison, the expressions of the four types of subunits decreased after DAPT was added. These results suggested that the inhibition of the NF‐κB signaling pathway was related to the intracellular domain of Notch1 (Figure 4A‐E). On the basis of this observation, we identified the expression level of NICD1 in the nucleus at 2 hours and showed that the expression level of the type four subunit was consistent with the significant difference (Figure 4F‐J).

### 3.5 IL‐1β‐ and TNF‐α‐promoted combination of NICD1 with p65 and p50

**Figure 5:**
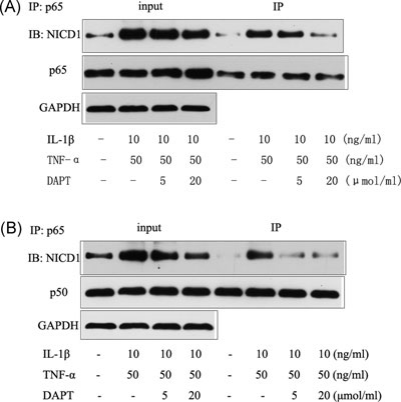
A, IP for combination of NICD1 with p65. B, IP for combination of NICD1 with p50. IP, immunoprecipitation

In hNPCs and under IL‐1β and TNF‐α induction, DAPT, as a specific inhibitor of the Notch signaling pathway, can inhibit the phosphorylation and acetylation of p65 as well as the nucleation of p65, P50, P52, and RelB. We hypothesized that the combination of NICD1 and NF‐κB subunits promoted the phosphorylation and acetylation of p65 as well as the nucleation of each subunit. We determined the combination of NICD1 with each subunit through IP. IL‐1β and TNF‐α increased the combination of NICD1 with p65 and p50, and the combination reduced after the reduction of NICD1 release (Figure 5A and 5B). These findings indicated that the activation of the NF‐κB p65 signaling pathway in hNPCs required NICD1 release.

### 3.6 Plasmid transfected 293t cells validated the NICD1 and p65 binding domains, NICD1, and p65 binding site in the ANK domain

**Figure 6:**
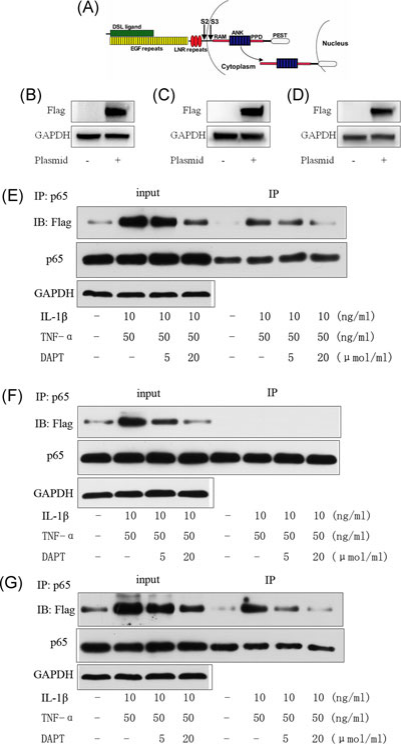
A, NICD1 Shearing site and structural domain diagram. Plasmid transfection of 293T cells: B, Overexpression of RAM+ANK‐Flag structural domain; C, Overexpression of RAM+TAD+PEST‐Flag structural domain (include CSL structural domain); D, Overexpression of ANK+TAD+PEST‐Flag structural domain. E, Under the induction of IL‐1β and TNF‐α, IP for 293T cells overexpress RAM+ANK‐Flag. F, Under the induction of IL‐1β and TNF‐α, IP for 293T cells overexpress RAM+TAD+PEST‐Flag structural domain. G, Under the induction of IL‐1β and TNF‐α, IP for 293T cells overexpress ANK+TAD+PEST‐Flag structural domain

We divided NICD1 into a three‐segment structural domain to build a plasmid: (a) overexpressed RAM+ANK structural domain; (b) overexpressed RAM+TAD+PEST structural domain (including the CSL domain); and (c) overexpressed ANK+TAD+PEST structural domain (Figure 6A). After three‐segment structural domain plasmids were transfected into 293T cells, Western blot displays that the transfection succeeded with the NICD1 structural domain (Figure 6B‐D).

Three of the expression plasmids transfected 293T cells to validate the NICD1 structural domain binding to p65. Under IL‐1β and TNF‐α induction, the transfected RAM+ANK‐Flag and ANK+TAD+PEST‐Flag plasmids still possessed NICD1 combined with p65 (Figure 6E and 6G). After the RAM+TAD+PEST‐Flag structural domain (including the CSL structural domain) plasmid was overexpressed under IL‐1β and TNF‐α induction, the 293T cells could not undergo NICD1 and p65 binding (Figure 6F). Under IL‐1β and TNF‐α induction, ANK is the binding site of NICD1 and p65.

### 3.7 Lentiviral vector with the deletion of the ANK zone overexpression and carrier transfection in hNPCs confirms that the ANK domain is the binding site of NICD1 and p65

**Figure 7:**
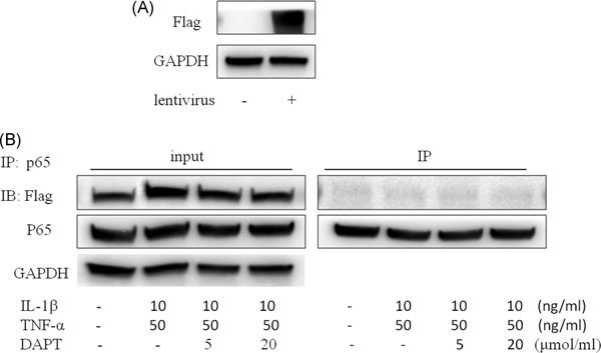
A, Immunoblot of hNPCs transfected with a lentivirus overexpressing RAM+TAD+PEST‐Flag structural domain. B, IP for P65 and NICD1 under the induction of IL‐1β and TNF‐α. 47 × 28 mm. hNPCs, human nucleus pulposus cells; IP, immunoprecipitation

The ANK domain found in the 293T cells is the binding site of NICD1 and p65. We used a lentiviral vector with the overexpression of the RAM+TAD+PEST‐Flag structural domain in hNPCs. IP revealed that NICD1 in the absence of the ANK region could not combine with p65 induced by IL‐1β and TNF‐α in hNPCs (Figure 7A and 7B).

## 4 DISCUSSION

The NF‐κB signaling pathway is activated in intervertebral disc degeneration and considered the main pathway of inflammation‐induced intervertebral disc degeneration.^14^ NF‐κB is the major and common activation signaling pathway of IL‐1β and TNF‐α. NF‐κB has five types of subunits: p65 (RelA), P50, P52, RelB, and Rel (cRel). They form a homologous or heterologous dimer in cells, in which p65‐P50 is widely distributed. They also play a major role in the activation of classical NF‐κB signaling pathways. In the resting state of the NF‐κB signaling pathway, the combination of a subunit and an inhibitory factor forms IκB, and a homologous or heterologous dimer is released after ubiquitination and phosphorylation occur under the action of Il‐1β, TNF‐α, and LPs. A downstream effect is then produced by nucleation.^15^

In the activation of NF‐κB and Notch signaling pathways in many inflammation‐related diseases,^9^, ^10^, ^11^, ^12^ a series of activation processes may occur in intervertebral disc degeneration. The NF‐kB signaling pathway activates the downstream effect after several dimers are stimulated, and the classical Notch signaling pathway requires the release of the intracellular domain of a Notch receptor.^16^ However, whether inflammation associated with nucleus pulposus cells can be regulated through the Notch signaling pathway remains unknown. As such, we developed a model of inflammatory factor‐induced intervertebral disc and nucleus pulposus cell degeneration using DAPT and used it in this study. In a previous study, DAPT reversed the IL‐1β‐ and TNF‐α‐induced degeneration of the intervertebral disc and nucleus pulposus cells, whose IL‐1β‐ and TNF‐α‐induced degeneration was likely controlled by DAPT through the modulation of the NF‐κB signaling pathway. NF‐κB is activated by polymer nucleation, and such activation is terminated in the nucleus of an NF‐κB subunit because the distribution and effect of p65‐p50 are extensive.^17^ As such, we mainly examined the p65‐p50 dimer. We found that DAPT could inhibit p65 phosphorylation, indicating that DAPT prevented NF‐κB activation. DAPT also blocks the acetylation of p65, which triggers transcription factors after the p65‐p50 dimer is phosphorylated, and histone acetyltransferases in the nucleus of p65 are acetylated. P65 acetylation results in reduced adhesion to IκBα and the continuous activation of NF‐κB signaling pathways. However, intracellular histone deacetylation enzyme causes p65 acetylation, thereby enhancing the p65 and IκBα binding ability to transport the complex cell nucleus via a nuclear transport protein (CRMI).^18^ This system exists in organisms and acts as an effective device to prevent the activation of the NF‐κB signal. Therefore, DAPT not only suppresses NF‐κB activation but also terminates the activation of the existing NF‐κB. We examined the expression levels of NF‐κB subunits in the nucleus and confirmed that DAPT could inhibit the IL‐1β‐ and TNF‐α‐induced nucleation of p65, P50, p52, and RelB.

DAPT inhibits the phosphorylation and acetylation of p65 as well as the nucleation of related subunits. IL‐1β and TNF‐α also induce NICD1 release in human myeloid cells. As such, we speculated that NICD1 could be combined with NF‐κB subunits or other effector proteins. IP also revealed that the combination of NICD1 and p65 induced by IL‐1β and TNF‐α was increased, thereby confirming our assumption that NICD1 and p65 were combined in hNPCs to stimulate the phosphorylation and acetylation of p65.

The Notch1 receptor is a 300 kDa single transmembrane protein composed of 2753 amino acid residues, a carboxyl terminal in the cytoplasm, and an amino terminal outside the cytoplasm.^19^ Notch receptors release the active intracellular part after shearing and participate in the activation of the downstream signaling pathway. The sheared portion is located in the transmembrane area. The Notch1 intracellular region contains the following structures: (a) RAM structural domain (high affinity CSL binding site), whose main function is to combine with CBF1/RbJ‐jk, such as in downstream signaling proteins; (b) 6 cdc10 (cell division cycle gene 10) repeat area/anchor protein (ANK), whose function with Deltex, the mastermind, and other downstream signals are combined to modify the pathway; (c) is located at the two ends of the anchor protein and two nuclear localization signals; (d) TAD; and (e) PEST structural domain (praline, glutamate, serine, threonine‐rich domain), the area rich in proline (P), glutamic acid (E), serine (S) and threonine (T), mainly involved in Notch protein degradation.^20^

**Figure 8:**
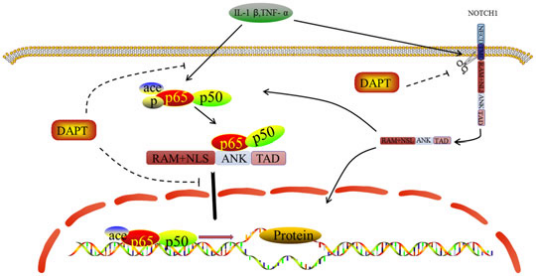
The cross talk between Notch and NF‐κB signaling pathway

We hypothesized that the RAM or ANK zone of NICD1 is the binding site of p65. As such, the design of the overexpressed plasmids involved NICD1 with three segments: (a) RAM+ANK structural domain, (b) RAM+TAD+PEST structural domain (including the CSL structural domain), and (c) ANK+TAD+PEST structural domain. We used 293T cells to verify the binding site of NICD1 and p65, and our results confirmed that the ANK area is a combined site of NICD1 and p65. Therefore, we used a slow virus to pack the ANK area, to remove the carrier‐transfected hNPCs and to verify the association of NICD1 with the binding site of p65. IP confirms that the ANK area is the NICD1 and p65 combined site. Thus, our results supported our previous observations that the ANK area is the binding site of Notch1 NICD1 for p65. NICD1 activates p65 phosphorylation and acetylation by combining ANK areas with p65 to adjust the activation state of the signaling pathway in the domain (Figure 8).

This study was limited by its inability to address problems associated with intervertebral disc medication and drug penetration difficulties.^21^ Oral administration easily causes toxic side effects on tissues, and local puncture to administer drugs is invasive, has a complex operation and has limited application.^22^ This study also failed to deeply examine the ANK region combining with the NF‐κB subunits. In fact, we found the combination phenomenon of NICD1 and P50, but such combination could not be ruled out because the combination between p65 and p50 could induce the IP of p50. However, we should note that the direct combination of NICD1 with P50 leads to coprecipitation. Therefore, follow‐up research should be performed.

This study provided a basis for further research on intervertebral disc degeneration under the action of inflammatory factors. Previous studies on the activation of the NF‐kB signaling pathway were complicated. The NF‐κB signaling pathway is also complex, and many activation mechanisms, such as Wnt and PI3K, are involved in the signaling pathway.^23^, ^24^ Therefore, the specificity of the suppression of the NF‐kB signaling pathway to a target point is ineffective. Currently, studies have focused on the series activation of the NF‐κB signaling pathway and other signaling pathways, indicating that the NF‐κB signaling pathway cannot be limited to key nodes implicated in the activation of the NF‐κB signaling pathways in series with other signaling pathways. Although previous studies involved protein‐protein interactions, in‐depth structural domain studies have yet to be performed. Consequently, difficulties arise in specifically examining the targeted inhibition of the NF‐κB signaling pathway activation. Our results also provided insights into the activation of Notch and NF‐κB signaling pathways in series. However, the inhibition of NICD1 in the ANK region can prevent the activation of the NF‐κB signaling pathway.

## 5 CONCLUSIONS

The following conclusions can be drawn from the study: (a) DAPT reversed IL‐1β‐ and TNF‐α‐induced degeneration of intervertebral disks and human myeloid cells; (b) in hNPCs, DAPT suppressed p65 phosphorylation and acetylation by preventing the release of NICD1, thereby inhibiting the activation of the NF‐κB signaling pathway; (c) in hNPCs, p65 phosphorylation and acetylation should be combined with the ANK domain of NICD1.

## Supporting information


**Supplemental figures**

**Supplemental figures**

**Supplemental figures**

## References

[1] Patrick N , Emanski E , Knaub MA . Acute and chronic low back pain. Med Clin North Am. 2016;100:169‐181. 26614726 10.1016/j.mcna.2015.08.015

[2] Yang W , Yu XH , Wang C , et al. Interleukin‐1beta in intervertebral disk degeneration. Clin Chim Acta. 2015;450:262‐272. 26341894 10.1016/j.cca.2015.08.029

[3] Huang KY , Lin RM , Chen WY , Lee CL , Yan JJ , Chang MS . IL‐20 may contribute to the pathogenesis of human intervertebral disc herniation. Spine. 2008;33:2034‐2040. 18758357 10.1097/BRS.0b013e31817eb872

[4] Yurube T , Takada T , Suzuki T , et al. Rat tail static compression model mimics extracellular matrix metabolic imbalances of matrix metalloproteinases, aggrecanases, and tissue inhibitors of metalloproteinases in intervertebral disc degeneration. Arthritis Res Ther. 2012;14:R51. 22394620 10.1186/ar3764 PMC3446417

[5] Nerlich AG , Bachmeier BE , Schleicher E , Rohrbach H , Paesold G , Boos N . Immunomorphological analysis of RAGE receptor expression and NF‐kappaB activation in tissue samples from normal and degenerated intervertebral discs of various ages. Ann N Y Acad Sci. 2007;1096:239‐248. 17405935 10.1196/annals.1397.090

[6] Nasto LA , Seo HY , Robinson AR , et al. ISSLS prize winner: inhibition of NF‐kappaB activity ameliorates age‐associated disc degeneration in a mouse model of accelerated aging. Spine. 2012;37:1819‐1825. 22343279 10.1097/BRS.0b013e31824ee8f7 PMC3395770

[7] Ellman MB , Kim JS , An HS , et al. The pathophysiologic role of the protein kinase Cdelta pathway in the intervertebral discs of rabbits and mice: in vitro, ex vivo, and in vivo studies. Arthritis Rheum. 2012;64:1950‐1959. 22161873 10.1002/art.34337 PMC3307815

[8] Miele L . Notch signaling. Clin Cancer Res. 2006;12:1074‐1079. 16489059 10.1158/1078-0432.CCR-05-2570

[9] Jiao Z , Wang W , Guo M , et al. Expression analysis of Notch‐related molecules in peripheral blood T helper cells of patients with rheumatoid arthritis. Scand J Rheumatol. 2010;39:26‐32. 20132067 10.3109/03009740903124424

[10] Murea M , Park JK , Sharma S , et al. Expression of Notch pathway proteins correlates with albuminuria, glomerulosclerosis, and renal function. Kidney Int. 2010;78:514‐522. 20531454 10.1038/ki.2010.172 PMC3164583

[11] Aoyama T , Takeshita K , Kikuchi R , et al. gamma‐Secretase inhibitor reduces diet‐induced atherosclerosis in apolipoprotein E‐deficient mice. Biochem Biophys Res Commun. 2009;383:216‐221. 19345673 10.1016/j.bbrc.2009.03.154 PMC2929363

[12] Ito T , Allen RM , Carson WF , et al. The critical role of Notch ligand Delta‐like 1 in the pathogenesis of influenza A virus (H1N1) infection. PLoS Pathog. 2011;7:e1002341. 22072963 10.1371/journal.ppat.1002341 PMC3207886

[13] Pelle DW , Peacock JD , Schmidt CL , et al. Genetic and functional studies of the intervertebral disc: a novel murine intervertebral disc model. PLoS One. 2014;9:e112454. 25474689 10.1371/journal.pone.0112454 PMC4256369

[14] Zhongyi S , Sai Z , Chao L , Jiwei T . Effects of nuclear factor kappa B signaling pathway in human intervertebral disc degeneration. Spine. 2015;40:224‐232. 25494317 10.1097/BRS.0000000000000733

[15] Siomek A . NF‐kappaB signaling pathway and free radical impact. Acta Biochim Pol. 2012;59:323‐331. 22855720

[16] LaFoya B , Munroe JA , Mia MM , et al. Notch: a multi‐functional integrating system of microenvironmental signals. Dev Biol. 2016;418:227‐241. 27565024 10.1016/j.ydbio.2016.08.023 PMC5144577

[17] Sun SC . Non‐canonical NF‐kappaB signaling pathway. Cell Res. 2011;21:71‐85. 21173796 10.1038/cr.2010.177 PMC3193406

[18] Aurora AB , Biyashev D , Mirochnik Y , et al. NF‐kappaB balances vascular regression and angiogenesis via chromatin remodeling and NFAT displacement. Blood. 2010;116:475‐484. 20203265 10.1182/blood-2009-07-232132 PMC2913457

[19] Hussain M , Xu C , Ahmad M , et al. Notch signaling: linking embryonic lung development and asthmatic airway remodeling. Mol Pharmacol. 2017;92:676‐693. 29025966 10.1124/mol.117.110254

[20] Nam Y , Aster JC , Blacklow SC . Notch signaling as a therapeutic target. Curr Opin Chem Biol. 2002;6:501‐509. 12133727 10.1016/s1367-5931(02)00346-0

[21] Kepler CK , Ponnappan RK , Tannoury CA , Risbud MV , Anderson DG . The molecular basis of intervertebral disc degeneration. Spine J. 2013;13:318‐330. 23537454 10.1016/j.spinee.2012.12.003

[22] Wang S , Rui Y , Lu J , Wang C . Cell and molecular biology of intervertebral disc degeneration: current understanding and implications for potential therapeutic strategies. Cell Prolif. 2014;47:381‐390. 25112472 10.1111/cpr.12121 PMC6495969

[23] Ma B , Hottiger MO . Crosstalk between Wnt/beta‐catenin and NF‐kappaB signaling pathway during inflammation. Front Immunol. 2016;7:378. 27713747 10.3389/fimmu.2016.00378 PMC5031610

[24] Iyer AKV , Azad N , Talbot S , et al. Antioxidant c‐FLIP inhibits Fas ligand‐induced NF‐kappaB activation in a phosphatidylinositol 3‐kinase/Akt‐dependent manner. J Immunol. 2011;187:3256‐3266. 21856935 10.4049/jimmunol.1002915 PMC3169770

