# Supplementary figures and images for: Gamma‐secretase inhibitor suppressed Notch1 intracellular domain combination with p65 and resulted in the inhibition of the NF‐κB signaling pathway induced by IL‐1β and TNF‐α in nucleus pulposus cells
Source: J Cell Biochem. 2018 Oct 26;120(2):1903–15. doi: 10.1002/jcb.27504 (PMC6587483; doi:10.1002/jcb.27504)

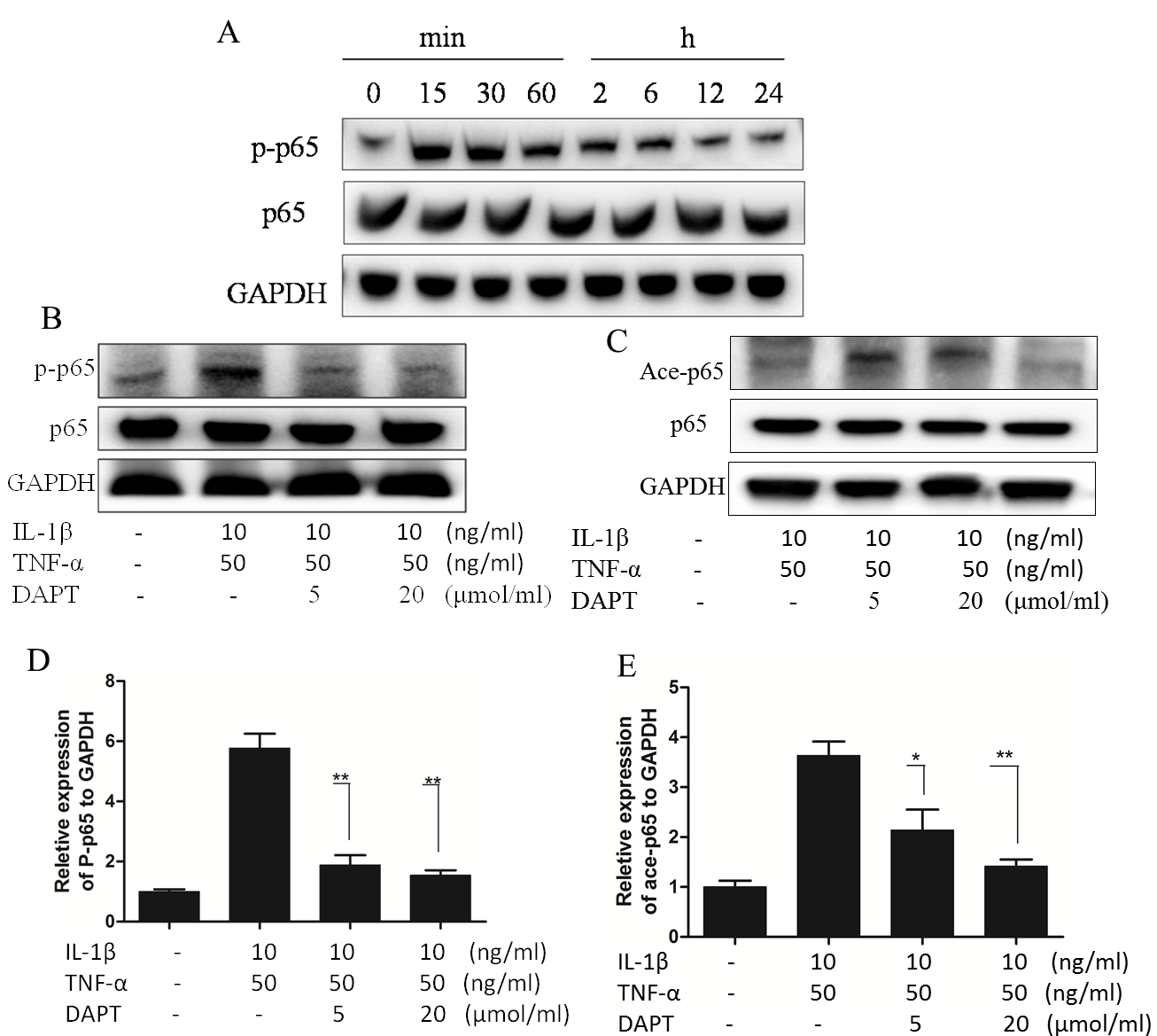

Supplement: Supplementary file 1 — Supplemental figures [file JCB-120-1903-s001.tif]

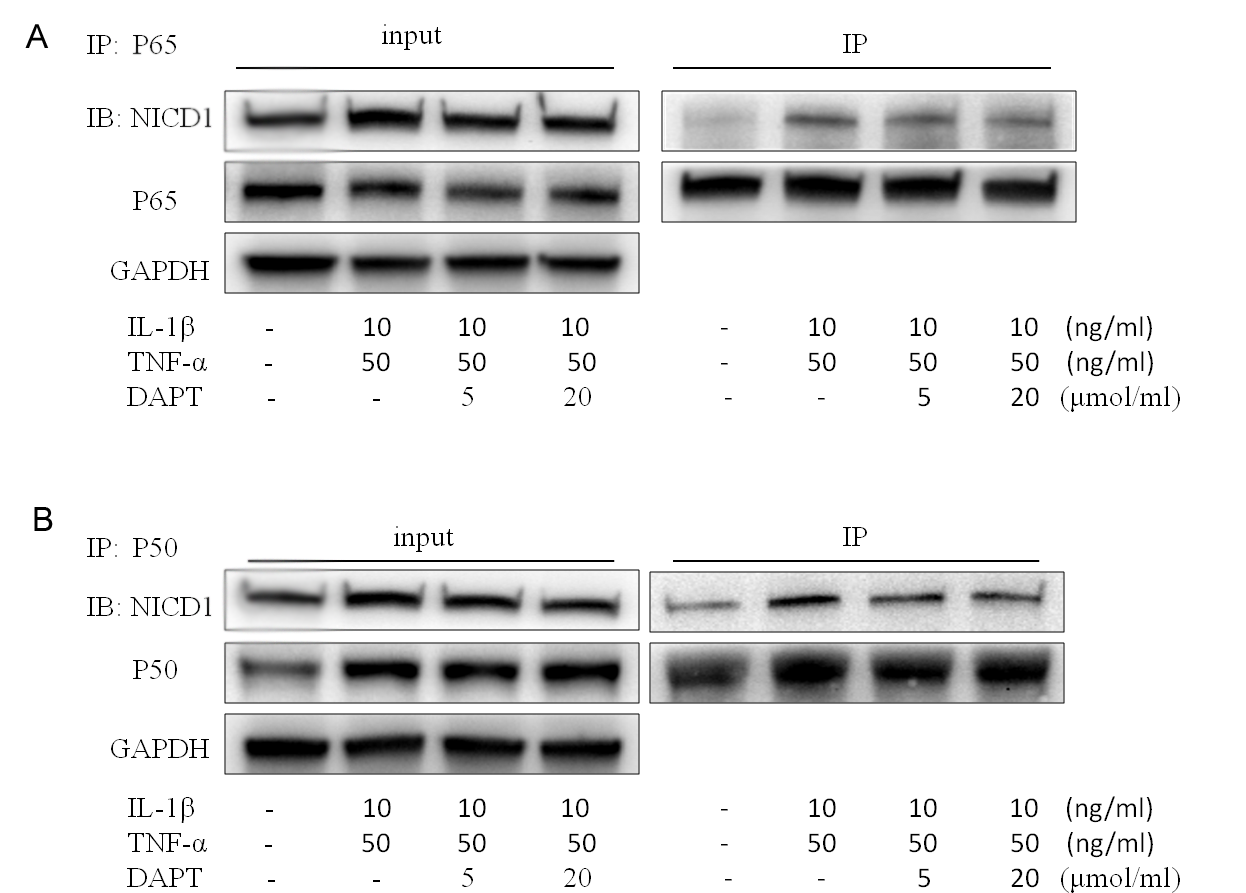

Supplement: Supplementary file 2 — Supplemental figures [file JCB-120-1903-s002.tif]

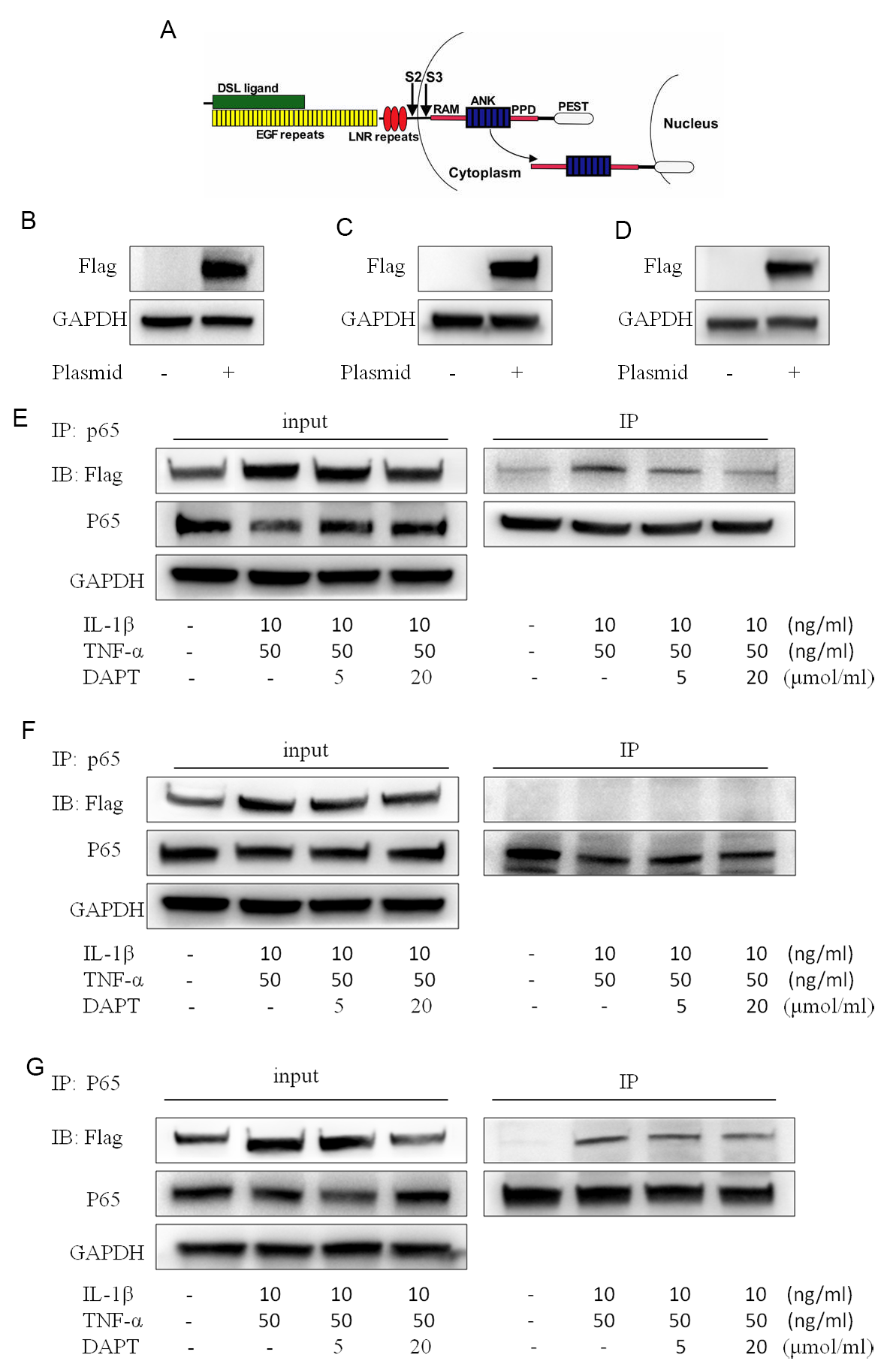

Supplement: Supplementary file 3 — Supplemental figures [file JCB-120-1903-s003.tif]
